# Supplementary material for: Impact of Ce/Zr Ratio in the Nanostructured Ceria and Zirconia Composites on the Selective CO2 Adsorption
Source: Nanomaterials (Basel). 2023 Aug 26;13(17):2428. doi: 10.3390/nano13172428 (PMC10490239; doi:10.3390/nano13172428)
Supplement: Supplementary file 1 [file nanomaterials-13-02428-s001.zip › nanomaterials-2546083-supplementary.pdf]

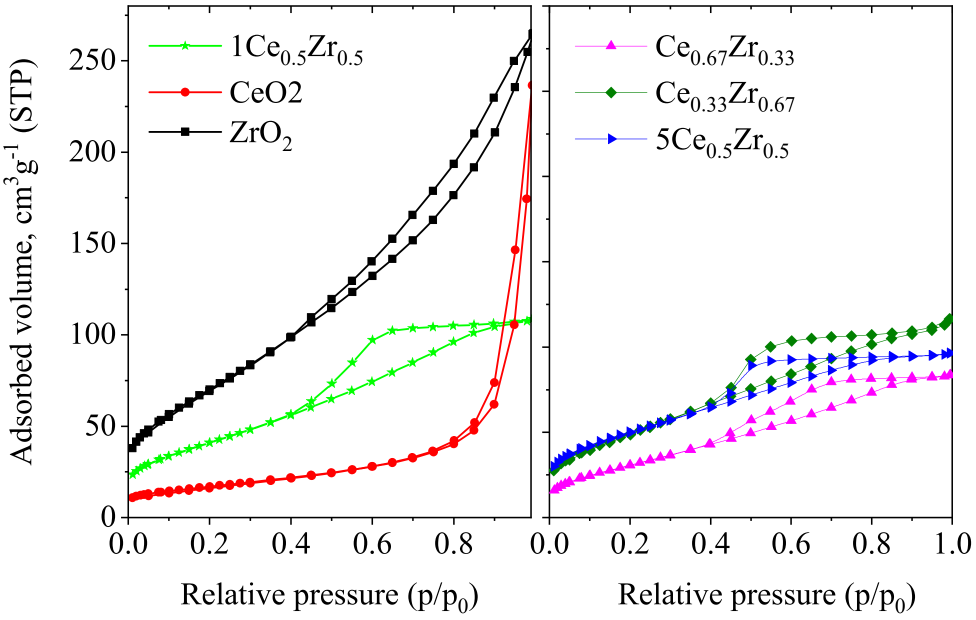

Figure S1. Nitrogen physisorption isotherms of the studied CeO_2, ZrO_2 and CeO_2/ZrO_2 mixed oxide materials

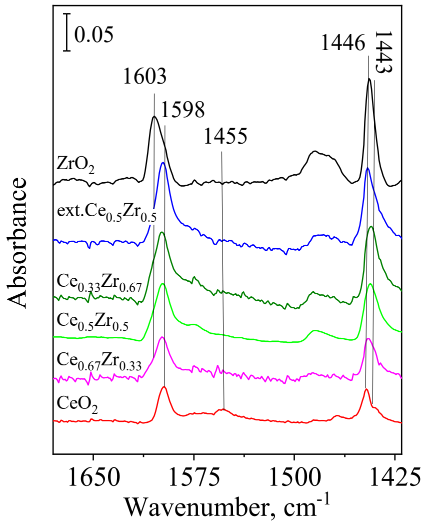

Figure S2. FT-IR spectra of adsorbed pyridine on the studied adsorbents. Py (6 mbar) was adsorbed on 300 °C dehydrated samples at 100 °C and desorbed at 100 °C in high vacuum.

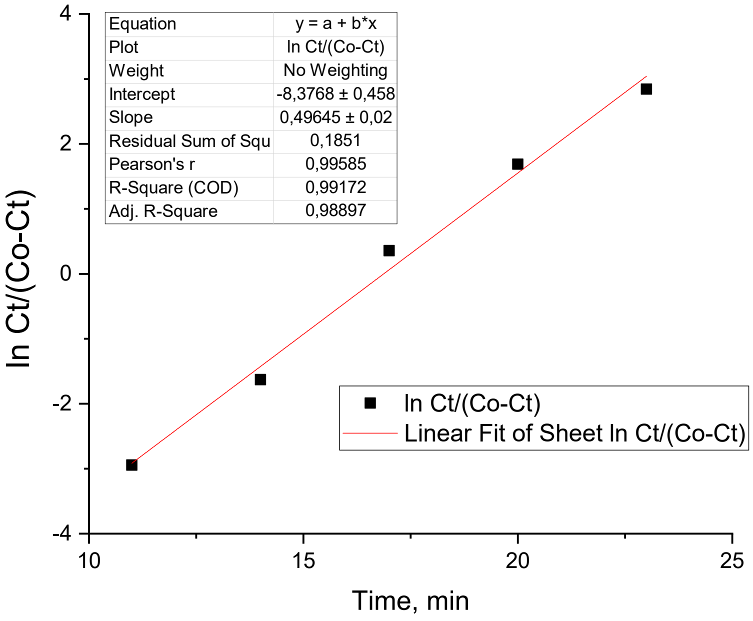

Figure S3. Fitting of experimental data on kinetic model at 0 ◦C
